# Efficacy of sacubitril/valsartan versus olmesartan in Japanese patients with essential hypertension: a randomized, double-blind, multicenter study

**DOI:** 10.1038/s41440-021-00819-7

**Published:** 2022-01-21

**Authors:** Hiromi Rakugi, Kazuomi Kario, Masako Yamaguchi, Takayoshi Sasajima, Hiromi Gotou, Jack Zhang

**Affiliations:** 1 Department of Geriatric and General Medicine, Osaka University Graduate School of Medicine, Osaka, Japan; 2 Division of Cardiovascular Medicine, Department of Medicine, Jichi Medical University School of Medicine, Tochigi, Japan; 3 Novartis Pharma K. K, Tokyo, Japan; 4 Novartis Pharmaceuticals Corporation, East Hanover, NJ USA

**Keywords:** Angiotensin receptor neprilysin inhibitor, Japanese, Olmesartan, Sacubitril/valsartan, Systolic hypertension

## Abstract

This phase III study assessed the efficacy and safety of sacubitril/valsartan compared with those of olmesartan in Japanese patients with essential hypertension. Patients (*n* = 1161, aged ≥20 years) with mild to moderate hypertension (mean sitting systolic blood pressure [msSBP] ≥150 to <180 mmHg) were randomized to receive sacubitril/valsartan 200 mg (*n* = 387), sacubitril/valsartan 400 mg (*n* = 385), or olmesartan 20 mg (*n* = 389) once daily for 8 weeks. The primary assessment was a reduction in msSBP from baseline with sacubitril/valsartan 200 mg vs. olmesartan 20 mg at Week 8. Secondary assessments included msSBP reduction with sacubitril/valsartan 400 mg vs. olmesartan at Week 8 and reductions in mean sitting diastolic blood pressure (msDBP), mean sitting pulse pressure (msPP), and overall blood pressure (BP) control rate for all treatment groups at Week 8. Sacubitril/valsartan 200 mg provided a significantly greater reduction in msSBP from baseline than olmesartan at Week 8 (between-treatment difference: −5.01 mmHg [95% confidence interval: −6.95 to −3.06 mmHg, *P* < 0.001 for noninferiority and superiority]). Greater reductions in msSBP with sacubitril/valsartan 400 mg vs. olmesartan, as well as in msDBP and msPP with both doses of sacubitril/valsartan *vs*. olmesartan (*P* < 0.05 for all), were also observed. Patients treated with sacubitril/valsartan achieved an overall higher BP control rate. The safety and tolerability profiles of sacubitril/valsartan were generally comparable to those of olmesartan. The adverse event rate with sacubitril/valsartan was not dose-dependent. Treatment with sacubitril/valsartan was effective and provided superior BP reduction, with a higher proportion of patients achieving target BP goals than treatment with olmesartan in Japanese patients with mild to moderate essential hypertension.

## Abstract (web summary)

Treatment with sacubitril/valsartan is effective and provided superior blood pressure (BP) reductions with a higher proportion of patients achieving target BP goals versus olmesartan in Japanese patients with mild-to-moderate essential hypertension.

## Graphical abstract

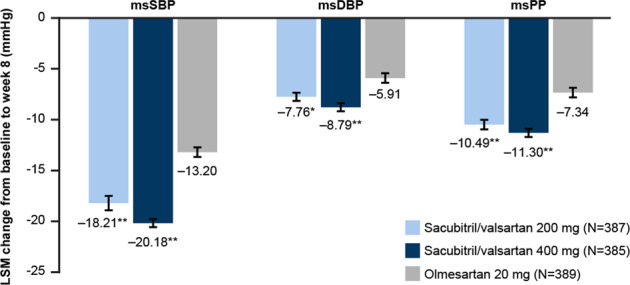

## Introduction

Hypertension is a major risk factor for cardiovascular (CV) diseases, and its prevalence is rapidly increasing in Asia, including Japan [1, 2]. An estimated 43 million individuals in Japan are affected by hypertension, and the incidence of hypertension is expected to increase in view of the growing elderly population [3, 4]. Despite the availability of a large number of recommended treatment options, blood pressure (BP) control remains inadequate in the majority of Japanese patients [3, 5], especially in elderly patients, generally because of a lack of systolic blood pressure (SBP) control. New therapeutic options that target the underlying pathophysiology of systolic hypertension would therefore be beneficial in reducing the burden of this condition.

Sacubitril valsartan sodium hydrate (LCZ696, sacubitril/valsartan), a first-in-class angiotensin receptor neprilysin inhibitor (ARNI) that provides simultaneous neprilysin inhibition and angiotensin II receptor-1 blockade, has been approved for the treatment of heart failure with reduced ejection fraction (HFrEF) in several countries worldwide in view of its superior benefits over enalapril demonstrated in the PARADIGM-HF trial. Sacubitril/valsartan has been recently approved in Japan for the treatment of chronic heart failure [6–12]. The recent expansion of the indication for sacubitril/valsartan in the United States also allows the treatment of adults with a left ventricular ejection fraction below normal [13].

Neprilysin inhibition increases circulating levels of natriuretic peptides (NPs), which promote natriuresis, diuresis, vasodilation, and endothelial permeability and inhibit the renin-angiotensin-aldosterone system (RAAS), the sympathetic nervous system (SNS), aldosterone secretion, and fibrosis; these actions confer cardiac, vascular, and renal protection [14–16]. Sacubitril/valsartan has demonstrated superior reductions in systolic and pulse pressures in previous studies in both Western and Asian patients with mild to moderate hypertension [7, 17] compared with valsartan [7] and placebo [17], respectively. Long-term use of sacubitril/valsartan provided significant BP reductions from baseline in Asian patients with mild to moderate hypertension [18]. The efficacy and safety of sacubitril/valsartan has previously been demonstrated in Japanese patients with severe hypertension [19] and in patients with hypertension and renal impairment without a decline in renal function [20].

Olmesartan is a commonly prescribed angiotensin receptor blocker (ARB) for the management of systolic hypertension among Asians. Moreover, olmesartan was considered the most potent ARB when the current study was designed [21, 22], while more recent evidence suggests that olmesartan has safety and efficacy profiles that are similar to those of other ARBs [23–25].

Sacubitril/valsartan possesses activities of both angiotensin receptor blocking and neprilysin inhibition [26]; it is expected to demonstrate enhanced antihypertensive effects compared to an ARB. Previous studies have also demonstrated superior benefits of sacubitril/valsartan over olmesartan in reducing BP in patients with mild to moderate hypertension [27, 28]. However, the efficacy and safety of sacubitril/valsartan compared with those of olmesartan are not well established in Japanese patients with hypertension. Therefore, in the present phase III study, we assessed the BP-lowering efficacy and safety of sacubitril/valsartan in Japanese patients with mild to moderate essential hypertension and compared them with those of olmesartan.

## Methods

### Study design and patients

This was a multicenter, randomized, double-blind, parallel-group, and active-controlled study (Supplementary Fig. S1) conducted between June 2012 and April 2013 in Japan. This study is registered with ClinicalTrials.gov (identifier: NCT01599104).

Male or female Japanese patients aged ≥20 years with either treated or untreated mild to moderate systolic hypertension were eligible for this study. Elderly patients (≥ 65 years) were recruited to make up ~30% of the study population. Treated patients, defined as patients having a history of hypertension receiving antihypertensive medications within 4 weeks prior to screening, with mean sitting systolic blood pressure (msSBP) ranging from ≥150 to <180 mmHg at randomization and ≥140 to <180 mmHg at the visit immediately before randomization, were included. Untreated patients, defined as either (1) newly diagnosed hypertensive patients who had never taken any antihypertensive medications or (2) patients having a history of hypertension who had not been taking any antihypertensive medications for at least 4 weeks prior to screening and had msSBP ranging from ≥150 to <180 mmHg at both screening and randomization, were included.

Patients with severe hypertension (mean sitting diastolic blood pressure [msDBP] ≥110 mmHg or msSBP ≥180 mmHg) or secondary forms of hypertension were excluded from the study. For patient safety, the study also excluded patients with a history of angioedema, stroke or transient ischemic cerebral attack, myocardial infarction, coronary bypass surgery, or any percutaneous coronary intervention during the 12 months prior to screening. Pregnant or nursing mothers and women of childbearing potential were also excluded.

Following screening, prior antihypertensive medication was stopped, or the dose was tapered, and all eligible patients entered a single-blind, placebo run-in period of 2–4 weeks. Patients who successfully completed the run-in and met entry criteria were randomized in a 1:1:1 ratio to double-blind treatment with sacubitril/valsartan 200 mg, sacubitril/valsartan 400 mg (uptitrated following 1 week of 200 mg), or olmesartan 20 mg once daily for 8 weeks. The dose of the active comparator, olmesartan 20 mg, is the commonly prescribed daily dose in Japan. All eligible patients were randomized using an Interactive Web Response System (IWRS). The identity of the treatments was concealed by using study drugs that were identical in packaging, labeling, administration schedule, and appearance. During the treatment period, trial participants were instructed to take two tablets and one capsule (the study drugs or matching placebo), with or without food, in the morning and at approximately the same time each day during the course of the study.

The study protocol was reviewed and approved by the institutional review board or independent ethics committee of each participating center. The study was conducted in accordance with the ICH Harmonized Tripartite Guidelines for Good Clinical Practice (with applicable local regulations) and the ethical principles in the Declaration of Helsinki. All patients provided written informed consent before the initiation of any study procedure.

### Efficacy assessments

The primary assessment was testing the hypotheses of the noninferiority and superiority of sacubitril/valsartan 200 mg compared with olmesartan 20 mg in msSBP reductions from baseline at Week 8. Secondary assessments included reductions in msSBP from baseline with sacubitril/valsartan 400 mg compared with olmesartan at Week 8; msDBP and mean sitting pulse pressure (msPP) from baseline at Week 8; the proportion of patients achieving successful BP control (msSBP < 140 mmHg and msDBP < 90 mmHg), SBP control (msSBP < 140 mmHg), and DBP control (msDBP < 90 mmHg); and the safety and tolerability of sacubitril/valsartan (200 mg and 400 mg) compared with those of olmesartan. A subgroup analysis of the primary and secondary efficacy assessments (including msDBP, msPP, and overall BP control rate) by age group (<65 years and ≥65 years) was conducted. As a post hoc analysis, we assessed BP control rates according to the current Japanese Society of Hypertension (JSH) 2019 guideline (msSBP/msDBP < 130/80 mmHg for <75 years old and <140/90 mmHg for ≥75 years old). [4]

Clinical BP was measured at the trough using a validated automatic BP device (Omron HEM 7080IC). Blood pressure measurements were performed at screening through the end of the study at every visit. At each study visit, after the patient had been sitting for five minutes with their back supported and both feet placed on the floor, SBP and DBP were measured four times using the automatic BP monitor and an appropriate size cuff. Regular monitoring of clinical laboratory parameters was performed at a central laboratory, and regular assessments of vital signs and physical condition were conducted.

### Safety assessments

Safety assessments included the monitoring of all adverse events (AEs) and serious AEs (SAEs) during the conduct of this study (from screening to Week 8 visit), as well as the regular monitoring of vital signs (from screening to Week 8 visit) and full clinical laboratory tests (at screening, randomization, the Week 4 visit, and the Week 8 visit).

### Statistical analysis

The primary objective of this study was to compare the efficacy of sacubitril/valsartan 200 mg with that of olmesartan 20 mg by evaluating (i) the hypothesis of the noninferiority of sacubitril/valsartan 200 mg vs. olmesartan 20 mg for a decrease in msSBP from baseline and (ii) if the hypothesis of noninferiority was met, the hypothesis of the superiority of sacubitril/valsartan 200 mg over olmesartan 20 mg for a decrease in msSBP from baseline was tested.

The primary analysis of the change in msSBP from baseline was conducted using a one-way analysis of covariance (ANCOVA) model with treatment as a factor and baseline msSBP as a covariate.

Sacubitril/valsartan 200 mg was considered noninferior to olmesartan 20 mg if the result of the noninferiority test was statistically significant. The statistical test was made at a one-sided significance level of 0.025.

If the result of the noninferiority test was statistically significant, a superiority test was performed at a two-sided significance level of 0.05, where superiority for sacubitril/valsartan 200 mg vs. olmesartan 20 mg was considered to be achieved if the test was statistically significant in favor of sacubitril/valsartan. The continuous secondary endpoints were analyzed using a one-way ANCOVA model with the corresponding baseline value as a covariate. The BP control rates were analyzed using a logistic regression model with treatment as a factor and baseline value as a covariate. Summary statistics are presented for the age subgroup (aged > 65 years) for the primary and secondary variables. For both primary and secondary endpoints, missing values at Week 8 were imputed with the last postbaseline measurement as the last observation carried forward (LOCF), and analysis was performed for the full analysis set (FAS). For secondary endpoints, no multiplicity adjustment was made; therefore, statistical interpretations should be made with caution.

The frequencies of AEs, SAEs, and notable laboratory abnormalities were measured using the safety set (SAF) of all patients who received at least one dose of double-blind study medication.

### Sample size calculation

A sample size of 342 patients completing the study per group was targeted based on the primary efficacy variable and an SD of 14 mmHg to attain 90% power to detect a change of 3.5 mmHg at a two-sided significance level of 0.05 to test the hypothesis that sacubitril/valsartan 200 mg is superior to olmesartan 20 mg. Assuming a 10% drop-out rate, the total targeted sample size planned for randomization was 1140 patients. The sample size had ≥90% power for the noninferiority test, with a prespecified noninferiority margin of 2 mmHg, at a one-sided significance of 0.025 under the alternative hypothesis that sacubitril/valsartan 200 mg has a greater msSBP reduction of ≥1.5 mmHg than the olmesartan 20-mg treatment group.

## Results

### Patients

**Fig. 1:**
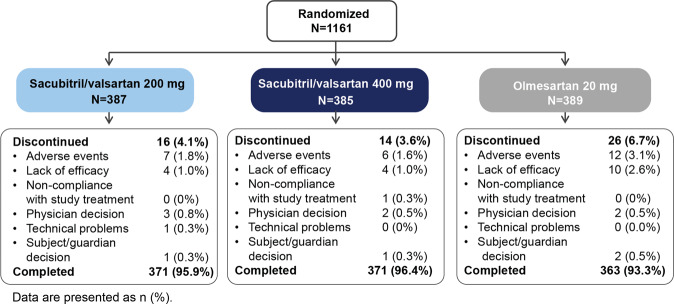
Patient disposition. Data are presented as n (%).

Of the 1161 randomized patients, 1105 (95.2%) completed the study (Fig. 1). Common reasons for discontinuation were AEs (2.2%) and a lack of efficacy (1.6%). Total discontinuation and discontinuation due to AEs or a lack of efficacy were more frequent in the olmesartan group than in the sacubitril/valsartan group. The majority of patients were male (70.5%), the mean age was 58.7 years, and approximately 32.9% of patients were elderly patients (aged ≥ 65 years). The mean duration of hypertension was 8.5 years, and 74.8% of the patients were treated with antihypertensive medications (Table 1).

**Table 1:** Patient demographics and baseline characteristics

Demographic/baseline variable	Sacubitril/valsartan 200 mg *N* = 387	Sacubitril/valsartan 400 mg *N* = 385	Olmesartan 20 mg *N* = 389	Total *N* = 1161
Age (years)	57.9 ± 10.9	58.7 ± 10.5	59.6 ± 10.5	58.7 ± 10.6
≥65 years, *n* (%)	119 (30.7)	127 (33.0)	136 (35.0)	382 (32.9)
Male, *n* (%)	264 (68.2)	268 (69.6)	286 (73.5)	818 (70.5)
Ethnicity, *n* (%)				
Japanese	387 (100.0)	385 (100.0)	389 (100.0)	1161 (100.0)
BMI (kg/m^2^)	25.4 ± 3.7	25.3 ± 3.9	25.6 ± 3.8	25.4 ± 3.8
Duration of hypertension history (years)	8.4 ± 6.8	8.3 ± 6.7	8.9 ± 7.2	8.5 ± 6.9
Antihypertensive medications, *n* (%)				
Treated*, *n* (%)	281 (72.6)	283 (73.5)	304 (78.1)	868 (74.8)
Untreated, *n* (%)	106 (27.4)	102 (26.5)	85 (21.9)	293 (25.2)
Having a history of hypertension**, *n* (%)	105 (27.1)	101 (26.2)	84 (21.6)	290 (25.0)
Newly diagnosed with hypertension^#^, *n* (%)	1 (0.3)	1 (0.3)	1 (0.3)	3 (0.3)
Diabetes, *n* (%)	29 (7.5)	35 (9.1)	43 (11.1)	107 (9.2)
eGFR group (mL/min/1.73 m^2^)				
30 ≤ eGFR <60, *n* (%)	90 (23.3)	113 (29.4)	131 (33.7)	334 (28.8)
60 ≤ eGFR < 90, *n* (%)	275 (71.1)	250 (64.9)	242 (62.2)	767 (66.1)
eGFR ≥ 90, *n* (%)	22 (5.7)	22 (5.7)	16 (4.1)	60 (5.2)
msSBP, mmHg	157.7 ± 6.9	158.4 ± 7.3	157.6 ± 6.8	157.9 ± 7.0
<65 years	157.56 ± 7.0	157.53 ± 6.7	157.03 ± 6.3	NA
≥65 years	158.01 ± 6.8	160.22 ± 8.2	158.78 ± 7.4	NA
msDBP, mmHg	94.3 ± 9.4	94.8 ± 9.8	93.8 ± 9.7	94.3 ± 9.6
<65 years	97.45 ± 7.9	97.94 ± 8.2	97.76 ± 7.2	NA
≥65 years	87.08 ± 8.6	88.35 ± 9.6	86.48 ± 9.3	NA
msPP, mmHg	63.4 ± 10.3	63.6 ± 11.3	63.8 ± 11.1	63.6 ± 10.9
<65 years	60.11 ± 8.8	59.59 ± 9.5	59.27 ± 8.5	NA
≥65 years	70.94 ± 9.3	71.87 ± 10.2	72.31 ± 10.5	NA

Data are presented as mean ± standard deviation unless specified

*BMI* body mass index, *DBP* diastolic blood pressure, *eGFR* estimated glomerular filtration rate, *ms* mean sitting, *NA* not available, *PP* pulse pressure, *SBP* systolic blood pressure

*Defined as patients having a history of hypertension receiving antihypertensive medications within 4 weeks prior to screening, with mean sitting systolic blood pressure (msSBP) ranging from ≥150 to <180 mmHg at randomization and ≥140 to <180 mmHg at the visit immediately before randomization

**Defined as patients having a history of hypertension who had not been taking any antihypertensive medications for at least 4 weeks prior to screening, and had msSBP ranging from ≥150 to <180 mmHg at both screening and randomization

^#^Newly diagnosed hypertensive patients who had never taken any antihypertensive medications

### Efficacy

#### Clinic blood pressure

**Fig. 2:**
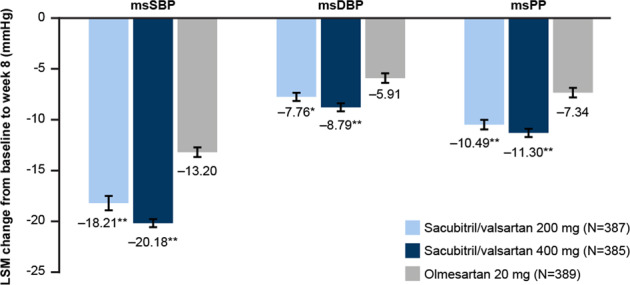
Change from baseline in msSBP, msDBP, and msPP at Week 8 (full analysis set). *P = 0.001; **P < 0.001 vs. olmesartan. LSM changes from baseline, standard errors, and *P* values calculated using ANCOVA with baseline as a covariate; error bars represent standard error; endpoint represents data at Week 8 or the last observation carried forward; *N* is the number of patients who had values at both baseline and the endpoint. ANCOVA analysis of covariance, BP blood pressure, LSM least squares mean, msDBP mean sitting diastolic BP, msPP mean sitting pulse pressure, msSBP mean sitting systolic BP

Sacubitril/valsartan 200 mg provided superior msSBP reduction from baseline over olmesartan at 8 weeks, with a between-treatment difference (95% confidence interval [CI]) of −5.01 mmHg (−6.95, −3.06; *P* < 0.001 for noninferiority and superiority) (Fig. 2). Similarly, sacubitril/valsartan 400 mg also provided greater msSBP reductions from baseline than olmesartan at Week 8, with a between-treatment difference (95% CI) of −6.97 (−8.92, −5.03; *P* < 0.001) mmHg. Similar results were observed for msDBP and msPP, with both doses of sacubitril/valsartan providing greater reductions from baseline to Week 8 than olmesartan (*P* ≤ 0.001 for all) (Fig. 2). Sacubitril/valsartan 400 mg provided numerically greater msSBP, msDBP, and msPP reductions than sacubitril/valsartan 200 mg.

In the subgroup analysis by age group, sacubitril/valsartan 200 mg and 400 mg showed numerically greater msSBP reductions from baseline to Week 8 than olmesartan in both elderly (aged ≥ 65 years) (−18.78, −19.52, and −11.51 mmHg, respectively), and nonelderly patients (aged < 65 years) (−17.94, −20.53, and −14.10 mmHg, respectively) (Table 2). The magnitude of the difference between the sacubitril/valsartan groups and the olmesartan group appeared to be greater in elderly patients. Similar results were observed for msDBP and msPP, with both doses of sacubitril/valsartan resulting in numerically greater reductions from baseline to Week 8 than olmesartan (Table 2). While olmesartan provided similar msPP reductions in elderly and nonelderly patients, both doses of sacubitril/valsartan resulted in a numerically greater reduction in msPP in elderly patients than in nonelderly patients.

**Table 2:** Change from baseline in msSBP, msDBP, and msPP at Week 8 endpoint in patients based on age (full analysis set)

Age group (years): < 65					Age group (years): ≥ 65			
Treatment group	*N*	Baseline (mean)	Endpoint (mean)	Change from baseline (mean [SD])	*N*	Baseline (mean)	Endpoint (mean)	Change from baseline (mean [SD])
msSBP								
Sacubitril/valsartan 200 mg	268	157.6	139.6	−17.9 (12.3)	119	158.0	139.2	−18.8 (12.8)
Sacubitril/valsartan 400 mg	258	157.5	137.0	−20.5 (13.0)	127	160.2	140.7	−19.5 (14.4)
Olmesartan 20 mg	253	157.0	142.9	−14.1 (14.6)	136	158.8	147.3	−11.5 (16.5)
msDBP								
Sacubitril/valsartan 200 mg	268	97.5	89.2	−8.3 (7.9)	119	87.1	80.5	−6.6 (7.0)
Sacubitril/valsartan 400 mg	258	97.9	88.3	−9.7 (8.8)	127	88.4	81.1	−7.3 (8.0)
Olmesartan 20 mg	253	97.8	91.0	−6.7 (8.4)	136	86.5	82.4	−4.1 (7.6)
msPP								
Sacubitril/valsartan 200 mg	268	60.1	50.5	−9.7 (8.5)	119	70.9	58.7	−12.2 (9.3)
Sacubitril/valsartan 400 mg	258	59.6	48.8	−10.8 (8.5)	127	71.9	59.6	−12.2 (11.1)
Olmesartan 20 mg	253	59.3	51.9	−7.4 (9.9)	136	72.3	64.9	−7.4 (11.9)

Endpoint represents data at Week 8 or last observation carried forward; *N* is the number of patients who had values at both baseline and endpoint

*BP* blood pressure, *msDBP* mean sitting diastolic BP, *msPP* mean sitting pulse pressure, *msSBP* mean sitting systolic BP, *SD* standard deviation

After 8 weeks of treatment, the mean change in pulse rate was 1.7, 0.7, and 0.9 bpm with sacubitril/valsartan 200 mg, sacubitril/valsartan 400 mg, and olmesartan 20 mg, respectively.

#### Blood pressure control

**Fig. 3:**
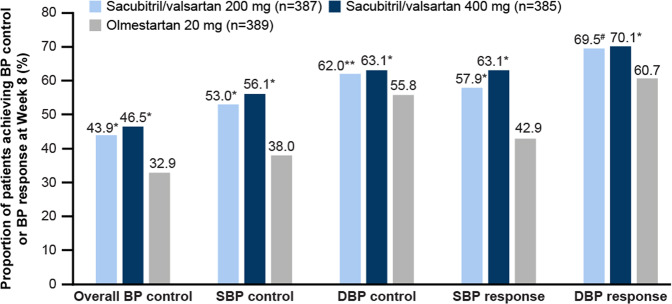
Proportion of patients who achieved overall BP, systolic and diastolic blood pressure control and response at the Week 8 endpoint (full analysis set). *P < 0.001 vs. olmesartan; **P = 0.019 vs. olmesartan; ^#^*P* = 0.002 vs. olmesartan. *P* values obtained from a logistic regression model with treatment as a factor and baseline value (msSBP for overall BP control and SBP control/response, msDBP for DBP control/response) as a covariate; *N* is the number of patients who have values at both baseline and the endpoint; endpoint represents data at Week 8 or the last observation carried forward. Overall, BP control was defined as msSBP/msDBP < 140/90 mmHg, SBP control as < 140 mmHg, DBP control as < 90 mmHg, SBP response as < 140 mmHg or a reduction from baseline ≥ 20 mmHg, and DBP response as <90 mmHg or a reduction from baseline ≥10 mmHg. BP blood pressure, DBP diastolic BP, ms mean sitting, SBP systolic blood pressure

At Week 8, a higher proportion of patients achieved overall BP control with both doses of sacubitril/valsartan than with olmesartan (Fig. 3). Similarly, the percentage of patients who achieved an SBP response (msSBP < 140 mmHg or a reduction from baseline ≥20 mmHg) and a DBP response (msDBP < 90 mmHg or a reduction from baseline ≥10 mmHg) was also greater in the sacubitril/valsartan treatment groups than in the olmesartan group (Fig. 3). As a post hoc analysis, we assessed the proportion of patients achieving BP control as defined by the 2019 JSH guideline (msSBP/msDBP <130/80 mmHg for <75 years old and <140/90 for ≥75 years old). A higher proportion of patients achieved BP control with sacubitril/valsartan 400 mg than with olmesartan 20 mg (21.8% vs. 14.4%, odds ratio [OR] 1.77, 95% CI [1.21, 2.59]). The proportion of patients who achieved BP control with sacubitril/valsartan 200 mg was 17.6%, vs. 14.4% in the olmesartan 20-mg group (OR 1.28, 95% CI [0.86, 1.89]).

### Safety

The adverse events occurring in ≥1.0% of patients in any treatment group are summarized in Table 3. Overall, the incidence of AEs and SAEs leading to discontinuation was numerically more frequent in patients treated with olmesartan than in those treated with either dose of sacubitril/valsartan. The incidence of AEs was comparable across age groups, with no age-specific trends observed.

**Table 3:** Number (%) of patients with adverse events ≥ 1% in any group during the 8-week treatment period (safety set)

Preferred term	Sacubitril/valsartan 200 mg *N* = 387, *n* (%)	Sacubitril/valsartan 400 mg *N* = 385, *n* (%)	Olmesartan 20 mg *N* = 389, *n* (%)
Any AEs	135 (34.9)	136 (35.3)	152 (39.1)
AE discontinuations	7 (1.8)	6 (1.6)	12 (3.1)
Drug-related AE discontinuations	2 (0.5)	2 (0.5)	4 (1.0)
SAEs	1 (0.3)	1 (0.3)	7 (1.8)
SAE discontinuations	1 (0.3)	1 (0.3)	4 (1.0)
Common AEs^†^			
Nasopharyngitis	48 (12.4)	47 (12.2)	46 (11.8)
Influenza	1 (0.3)	5 (1.3)	1 (0.3)
Back pain	0.0	5 (1.3)	1 (0.3)
Pharyngitis	5 (1.3)	4 (1.0)	1 (0.3)
Upper respiratory tract infection	2 (0.5)	4 (1.0)	2 (0.5)
Dermatitis contact	1 (0.3)	4 (1.0)	1 (0.3)
Headache	7 (1.8)	3 (0.8)	4 (1.0)
Blood creatine phosphokinase increased	4 (1.0)	3 (0.8)	4 (1.0)
Blood bilirubin increased	2 (0.5)	3 (0.8)	4 (1.0)
Dizziness	5 (1.3)	2 (0.5)	3 (0.8)
Cystitis	4 (1.0)	2 (0.5)	1 (0.3)
Diarrhea	2 (0.5)	1 (0.3)	5 (1.3)
Alanine aminotransferase increased	1 (0.3)	0.0	4 (1.0)
Hypertension	1 (0.3)	0.0	4 (1.0)
Hepatic function abnormal	0.0	0.0	5 (1.3)

AEs are sorted in descending frequency, as reported for sacubitril/valsartan 400 mg. A patient with multiple AEs within a primary system organ class is counted only once

*AE* adverse event, *SAE* serious adverse event

^†^≥1% in any treatment group

Nasopharyngitis was the most common AE, with a similar incidence in all groups. Only one event of hypotension (0.3% in the sacubitril/valsartan 400-mg group) was reported in this study. One event of angioedema (0.3%) was reported in the olmesartan group, but no angioedema was reported in either sacubitril/valsartan dose group. Generally, most of the AEs that occurred during the 8-week treatment period were infrequent, mild, and transient.

During the 8-week treatment period, nine patients experienced SAEs: 0.3% (1/387) in the sacubitril/valsartan 200-mg group (subarachnoid hemorrhage), 0.3% (1/385) in the sacubitril/valsartan 400-mg group (arteriosclerosis of the coronary artery and hepatobiliary disease), and 1.8% (7/389) in the olmesartan group (alanine aminotransferase increased, bile duct stone, cataract, cerebral infarction, osteoarthritis, radius fracture, and supraventricular tachycardia). No deaths occurred during the study.

During the 8-week treatment period, the mean changes from baseline in laboratory values were generally small. Potassium values >5.5 mmol/L were reported in 1.8% (7/387), 1.6% (6/385), and 0.8% (3/388) of patients treated with sacubitril/valsartan 200 mg and 400 mg and olmesartan 20 mg, respectively, with one patient in the sacubitril/valsartan 400-mg group experiencing a potassium value ≥6.0 mmol/L. In most of these patients, potassium levels returned to the normal range at the last study visit without any study medication disruption. Potassium values <3.5 mmol/L were reported in one patient treated with sacubitril/valsartan 200 mg, two treated with sacubitril/valsartan 400 mg, and one treated with olmesartan 20 mg. Blood urea nitrogen values >14.28 mmol/L and sodium values <130 mmol/L were reported for one patient in the sacubitril/valsartan 200-mg group; no patients in any treatment group showed a creatinine value >176.8 μmol/L. Mean decreases in uric acid were observed with sacubitril/valsartan 200 mg (−16.0 μmol/L) and 400 mg (−28.1 μmol/L), which were numerically greater than those observed with olmesartan 20 mg (−1.8 μmol/L).

## Discussion

In this phase III study, sacubitril/valsartan, a first-in-class ARNI, demonstrated superior reductions in clinical BP compared with olmesartan while showing comparable safety and tolerability in Japanese patients with hypertension. Sacubitril/valsartan 200 mg and 400 mg provided superior efficacy to olmesartan 20 mg in reducing msSBP by ~5 mmHg and 7 mmHg, respectively. These reductions in msSBP are clinically meaningful, as every 10-mmHg reduction in SBP is known to reduce the risk of major CV disease events by 20%, coronary heart disease by 17%, stroke by 27%, heart failure by 28%, and all-cause mortality by 13% [29]. These observations suggest that treatment with sacubitril/valsartan could substantially contribute to improving the clinical outcome of patients with hypertension.

Both sacubitril/valsartan doses were well tolerated, even in elderly patients. Moreover, no association was found between the AE rate and the dose of sacubitril/valsartan. The frequencies of discontinuations and SAEs were numerically lower in the sacubitril/valsartan groups than in the olmesartan group, and there were no deaths or reports of angioedema in either of the sacubitril/valsartan groups.

These results support the BP-lowering efficacy and safety of sacubitril/valsartan for the treatment of hypertension and are consistent with previous findings from studies in Asian patients [18], as well as findings from phase II and III studies in Western and Asian patients with mild to moderate essential hypertension [7, 17, 30]. Of particular note, the beneficial effects of sacubitril/valsartan on clinical measurements of SBP and PP were consistent across these studies. The subgroup analysis by age showed numerically greater differences in reductions from baseline in SBP and PP between sacubitril/valsartan and olmesartan in elderly patients than in nonelderly patients, without any safety concern. These observations are in accordance with an earlier study that demonstrated benefits of sacubitril/valsartan over olmesartan in elderly Asian patients with systolic hypertension [28]. Since increased SBP and PP have been identified as important CV risk factors in aging patients and lowering elevated SBP and PP has been shown to improve CV outcomes, our results suggest that treatment with sacubitril/valsartan may have a favorable effect on CV risk, irrespective of age group. A favorable benefit-risk profile of sacubitril/valsartan compared with enalapril in reducing heart failure hospitalization and mortality in all age groups was also observed in an analysis from the PARADIGM-HF study in patients with HFrEF. Sacubitril/valsartan was also superior to enalapril in preventing the deterioration of health-related quality of life across age ranges, even in the elderly group [31].

Asian populations may have differing levels of CV risk compared to Western populations. For example, a higher incidence of stroke than of coronary artery disease has been observed in Asian populations [32], and the association of raised BP with the risk of stroke appears to be stronger in Asian than Western populations [33]. In addition, Asian populations generally have a higher salt intake and are genetically more likely to have salt sensitivity than Western populations [34, 35]. High salt intake adversely impacts the ability of RAAS blockers such as olmesartan to lower BP. Due to its ability to inhibit neprilysin and through its multiple modes of action, sacubitril/valsartan is likely to be more potent in lowering BP, especially in populations with high salt intake [36]. Thus, the findings of this study in the Japanese population, in combination with the results of a similar study in the Chinese population [30], suggest promising clinical benefits for sacubitril/valsartan in Asian populations.

With aging, arteries gradually lose their elasticity, primarily due to collagen deposition and elastin depletion, resulting in increased peak SBP. Elevated PP is an indicator of vascular stiffness and is a strong predictor of risk associated with adverse CV events, including stroke, myocardial infarction, heart failure, CV disease, and CV mortality. Sacubitril/valsartan provided greater reductions in msPP than olmesartan in the current study, especially in elderly patients. These observations are further strengthened by the results of the PARAMETER study, which showed the superiority of sacubitril/valsartan 400 mg vs. olmesartan 40 mg in reducing clinical and ambulatory central aortic and brachial pressures in elderly patients with systolic hypertension and stiff arteries [37]. Such results suggest beneficial effects on arterial stiffness, an underlying cause of systolic hypertension. The reduction in PP observed in elderly patients in these studies could be due to a modulation of arterial wall elasticity, possibly brought about by antihypertrophic and antifibrotic properties of sacubitril/valsartan [38]. Simultaneous neprilysin inhibition and angiotensin II receptor-1 blockade have been shown to have antihypertrophic and antifibrotic effects in preclinical models, attenuating adverse tissue remodeling [38]. Such complementary neurohormonal modulation may provide an effective therapeutic approach to addressing arterial stiffness. In addition, in patients with HFrEF who were hospitalized for acute decompensated heart failure, sacubitril/valsartan treatment resulted in a rapid reduction in NT-proBNP levels, which was evident as early as one week [39]. This rapid and greater NT-proBNP reduction may also likely result in improved ventricular-vascular coupling. Sacubitril/valsartan may also augment the vasodilatory effects of natriuretic peptides, thereby reducing SBP and PP. Whether there are additional mechanistic factors contributing to the reduction of PP and SBP with sacubitril/valsartan and whether a longer treatment duration results in greater BP and PP reductions due to additional beneficial effects on arterial wall elasticity, particularly in elderly individuals, need further evaluation.

This study was limited to the assessment of peripheral BP that was measured at the clinic. Although reductions in SBP are associated with improved CV outcomes, the potential benefit of sacubitril/valsartan on 24-hour BP reduction and CV and renal outcomes in a hypertensive population remains to be elucidated.

## Conclusion

The results of this pivotal phase III study showed that treatment with sacubitril/valsartan is effective and generally well tolerated in Japanese patients with mild to moderate essential hypertension. Sacubitril/valsartan 200 mg and 400 mg once daily showed BP-lowering effects that were superior to those of olmesartan 20 mg.

## Supplementary information

Supplementary Figure S1
