# Supplementary material for: Efficacy of sacubitril/valsartan versus olmesartan in Japanese patients with essential hypertension: a randomized, double-blind, multicenter study
Source: Hypertens Res. 2022 Jan 21;45(5):824–33. doi: 10.1038/s41440-021-00819-7 (PMC9010294; doi:10.1038/s41440-021-00819-7)
Supplement: Supplementary file 1 — Supplementary Figure S1 [file 41440_2021_819_MOESM1_ESM.docx]

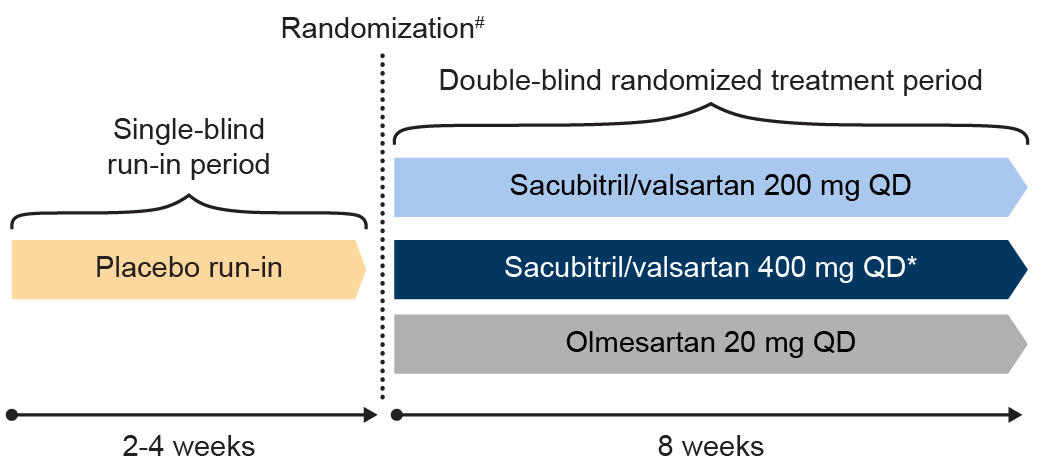


**Supplementary Figure S1.** Study design

*Titrated to sacubitril/valsartan 400 mg QD for 7 weeks following 1 week of sacubitril/valsartan 200 mg QD

^#^Patients who successfully completed treatment run-in and met BP criteria (msSBP ≥150 mmHg and <180 mmHg) were randomized in a 1:1:1 ratio to sacubitril/valsartan 200 mg or 400 mg QD or olmesartan 20 mg QD for 8 weeks.

msSBP, mean sitting systolic blood pressure; QD, once daily.
